# Prefrontal Cortex Glutamate Correlates with Mental Perspective-Taking

**DOI:** 10.1371/journal.pone.0003890

**Published:** 2008-12-08

**Authors:** Christiane Montag, Florian Schubert, Andreas Heinz, Jürgen Gallinat

**Affiliations:** 1 Department of Psychiatry and Psychotherapy, Charité University Medicine Berlin, Campus Mitte, Berlin, Germany; 2 Physikalisch-Technische Bundesanstalt Berlin, Berlin, Germany; James Cook University, Australia

## Abstract

**Background:**

Dysfunctions in theory of mind and empathic abilities have been suggested as core symptoms in major psychiatric disorders including schizophrenia and autism. Since self monitoring, perspective taking and empathy have been linked to prefrontal (PFC) and anterior cingulate cortex (ACC) function, neurotransmitter variations in these areas may account for normal and pathological variations of these functions. Converging evidence indicates an essential role of glutamatergic neurotransmission in psychiatric diseases with pronounced deficits in empathy. However, the role of the glutamate system for different dimensions of empathy has not been investigated so far.

**Methodology/Principal Findings:**

Absolute concentrations of cerebral glutamate in the ACC, left dorsolateral PFC and left hippocampus were determined by 3-tesla proton magnetic resonance spectroscopy (1H-MRS) in 17 healthy individuals. Three dimensions of empathy were estimated by a self-rating questionnaire, the Interpersonal Reactivity Index (IRI). Linear regression analysis showed that dorsolateral PFC glutamate concentration was predicted by IRI factor “perspective taking” (T = −2.710, p = 0.018; adjusted alpha-level of 0.017, Bonferroni) but not by “empathic concern” or “personal distress”. No significant relationship between IRI subscores and the glutamate levels in the ACC or left hippocampus was detected.

**Conclusions/Significance:**

This is the first study to investigate the role of the glutamate system for dimensions of theory of mind and empathy. Results are in line with recent concepts that executive top-down control of behavior is mediated by prefrontal glutamatergic projections. This is a preliminary finding that needs a replication in an independent sample.

## Introduction

Empathy is an essential element of human behavior. The multidimensional construct of empathy comprises various cognitive aspects (theory of mind, perspective-taking, cognitive empathy), which require an ability to adopt another person's perspective and to reason about complex mental states, as well as attributions of affective mental states mainly involving affective mirroring and a lesser degree of conscious inferencing [1]. Moreover, broader definitions of empathy include non-congruous reactions to the experience of others' emotional state, like sympathy and concern, but also self-oriented, mainly aversive feelings [2]. Dysfunctions of theory of mind and empathy have been seen as a basal feature of psychopathological syndromes in severe mental disorders like schizophrenia [3], [4] and autism [5]. Theory of mind deficits have been shown to bear trait characteristics and can be found in healthy relatives of schizophrenic patients [6], and high-schizotypal adults [7]. This may indicate a heritable component of empathic functioning related to individual differences in brain function.

Whilst brain imaging studies have shown the prefrontal and cingulate cortex activation to be linked to theory of mind and empathy [8], the role of neurotransmitter systems in this respect is largely unknown. Glutamatergic dysfunction has been suggested in various disorders marked by specific alterations of social reciprocity, like autism [9], [10] and schizophrenia [11], with research on the genetic underpinnings of these conditions currently focusing on the glutamatergic system [12], [11]. In animal experiments, glutamatergic neurotransmission, with particular reference to metabotropic glutamate receptor expression, has been implicated in social novelty discrimination [13] and considered to be a link between rearing conditions and adult prefrontal function [14].

In the human brain, abnormal in vivo glutamate concentrations have been determined by proton magnetic resonance spectroscopy (1H-MRS) in various disorders like schizophrenia [15], depression [16], [17] and autism [10], which in turn have been associated with dysfunctions of empathy. Although the association of glutamate measured in vivo with normal human behavior is currently investigated with great effort [18], the knowledge of the biological underpinnings of the different aspects of empathy is sparse. We investigated the relationship between self-rated dimensions of empathic responding, measured by the Interpersonal Reactivity Index (IRI) [2], and concentrations of cerebral glutamate determined by 3-tesla proton magnetic resonance spectroscopy (1H-MRS) The left dorsolateral prefrontal cortex (DLPFC), anterior cingulate cortex (ACC) with adjacent medial prefrontal cortex, and left hippocampus were chosen as regions of interest because of their dense glutamatergic innervation and their putative involvement in social cognitive processing [8], [20], [21].

## Results

The mean values were as follows, glutamate in the left ACC 15.1+/−1.3 (range 11.5–17.1) mmol/l, left prefrontal lobe 11.8+/−1.5 (range 8.8–13.6) mmol/l and left hippocampus 10.7+/−1.4 (range 7.7–12.5) mmol/l. Mean values for the IRI subscales were 25.9+/−3.7 (range 20–33) for ‘empathic concern’, 25.3+/−3.6 (range 18–31) for ‘perspective taking’ and 15.0+/−3.5 (range 8–20) for ‘personal distress’.

**Figure 1:**
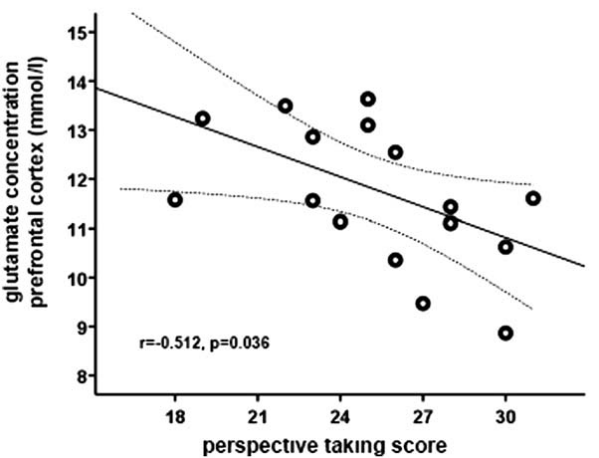
Correlation (Pearson) of prefrontal glutamate level and “perspective taking” score

Linear regression analysis showed that glutamate concentration in the DLPFC (dependent variable) was predicted by empathy factor “perspective taking” (T = −2.710, p = 0.018) but not by “empathic concern” (T = 1.078, p = 0.300), or “personal distress” (T = −1.313, p = 0.212). DLPFC glutamate level and “perspective taking” score showed a significant negative correlation (r = −0.512, p = 0.036, Pearson; **Figure 1**). Analyses of glutamate concentrations in the ACC and hippocampus using the empathy factors as predictors did not reveal significant effects. A conservative correction for multiple testing (three linear regression analyses) according to Bonferroni resulted in an adjusted alpha-level of 0.017 indicating a non-significant association between “perspective taking” and DLPFC glutamate level.

## Discussion

A negative association between left dorsolateral prefrontal concentrations of glutamate with ratings of perspective taking was observed as a preliminary finding. No associations were found for the other voxels of interest, left hippocampus and anterior cingulate cortex, nor with regard to the other subscales of the Interpersonal Reactivity Index. The association of DLPFC glutamate with perspective taking is compatible with functional brain imaging studies showing a role of the DLPFC in the cognitive regulation of empathy [1]. For instance, lateral prefrontal areas have been implicated in the representation of ‘other’ as opposed to ‘self’ [22]. Additionally, IRI cognitive subscores were found to be related to cognitive tasks that were based on the function of the DLPFC [23].

Recent evidence indicates that prefrontal glutamatergic neurotransmission together with dopamine activity is relevant for novelty seeking and goal-related behavior [24], [18]. Hippocampal glutamate concentration has also been implicated in the functional coupling between frontal cortical and hippocampal regions during stimulus processing [25]. The DLPFC entertains widespread interconnections within the social cognition network and is involved in executive functioning, maintaining cognitive flexibility and response inhibition [26], [27]. In line with this, inhibitory top-down control that suppresses prepotent self-perspective has been observed as an important factor with respect to perspective taking and theory of mind abilities [1], [28].

At first glance, the inverse direction of the correlation between prefrontal glutamate concentrations and perspective taking scores may seem counterintuitive, as in pathological conditions that are characterized by perspective taking deficits, like schizophrenia, a hypofunction of glutamatergic neurotransmission is implied. Therefore, in healthy persons the inverse correlation of prefrontal glutamate and perspective taking may be due to a nonlinear relationship between glutamate concentrations and prefrontal function. On the other hand, 1H-MRS data on glutamate/glutamine concentrations in schizophrenia is still inconsistent. Some studies report elevated levels of these metabolites in prefrontal and hippocampal voxels and global mental functioning has been associated with increased prefrontal absolute glutamate concentrations [29]. Own recent data revealed elevations of hippocampal glutamate concentrations in schizophrenic patients compared to healthy controls (Gallinat et al., in preparation). Moreover, clinical characteristics and illness duration seem to affect glutamine/glutamate levels in schizophrenic and high-risk individuals [15], [30], [31], [32] obscuring the nature of glutamate dysfunction in schizophrenia. Of interest, ketamine application in healthy subjects induces psychotic symptoms accompanied by a suppressed BOLD signal in the ventromedial prefrontal cortex [33], a key region of mental state reasoning. In general, it seems reasonable that also glutamate levels in healthy normal subjects are behaviorally relevant since glutamate concentrations have been shown to be associated with personality traits [18] and normal prefrontal function [25].

The lack of correlations between the emotional subscales of the IRI and DLPFC glutamate concentrations in the present study did not meet our expectation of DLPFC glutamate to affect top-down control in empathic reasoning. Furthermore, no evidence for a role of glutamate in the assessed IRI scores was observed in ACC and hippocampus, although these regions have been related to attention, self-monitoring and memory-based information processing as well as to personality traits not affected by attentional functioning [18]. Negative findings may be due mainly to a lack of statistical power or to the fact, that only a self-rating questionnaire was used to assess empathic response dispositions. The emotional subscales of the IRI focus on feelings of sympathy and altruism and may be more affected by socially desirable response tendencies than the cognitive subscale.

To our knowledge, this is the first investigation of the neurobiological basis of different dimensions of empathy using high field MRS. Our data suggest a possible involvement of cerebral glutamate in perspective-taking and add to findings that glutamate measured in vivo is relevant for human behavior and personality traits [18].

Beside the modest sample size, limitations of the present investigation concern the use of a single self-rating instrument to assess dimensions of empathy and the fact that matching control regions in the right hemisphere were not included in the measurements. As scanning time had to be restricted to a reasonable length, we decided to concentrate on brain regions involved in cognitive aspects of empathy. Moreover, our limited sample size did not allow to control for sex differences that must be assumed in empathic responding [33]. Results are therefore preliminary and should be replicated and extended with additional voxels as well as more objective measures of the multiple aspects of empathy in an independent sample.

## Materials and Methods

### Participants

Seventeen healthy individuals (29.7±6.8 years, 5 men, 12 women) without Axis I/II disorders (for details of the recruitment procedure see [18]) were investigated using MRS and the Interpersonal Reactivity Index (IRI) [2]. No subject from the current sample was investigated in one of our previous studies (e.g. [18]). The study was approved by the local ethics committee and all participants gave written informed consent.

### Magnetic resonance spectroscopy

MRS was carried out on a 3-tesla scanner (MEDSPEC 30/100, Bruker Biospin, Ettlingen, Germany) using an established method [34]. *T*_1_-weighted images were acquired using MDEFT (*T*_E_ = 3.8 ms, *T*_R_ = 20.53 ms; 128 contiguous slices, 1.5 mm thick; 1-mm inplane (*x*–*y*) resolution). After localized shimming magnetic resonance spectra were recorded from voxels including the left hippocampus (2×3×2 cm^3^), the anterior cingulate (2.5×4×2 cm^3^) and the left dorsolateral prefrontal cortex (2×2×2 cm^3^). For metabolite fitting, spectra were acquired from equal voxels in spherical metabolite phantoms (0.1 M metabolite, pH 7.2, 37°C). The PRESS (point resolved spectroscopy) sequence preceded by water suppression (3 Gauss CHESS pulses of 25.6 ms duration) was used throughout. For one metabolite spectrum 8 subspectra of 16 phase cycled scans each were recorded with *T*_R_ = 3 s. An echo time of 80 ms was chosen to obtain maximum selectivity for the glutamate C4 resonance. Before further processing, the 8 metabolite subspectra were corrected for eddy currents using water-unsuppressed spectra (n = 8), automatically corrected for frequency and phase shifts, and added together to give 128 averages. Spectral quantification was carried out using a time domain-frequency domain fitting procedure [34] that includes phantom basis spectra (see above) and prior knowledge, and involves background estimation by regularization. In the present spectra, the amplitudes of total choline, total creatine, N-acetylaspartate (NAA), glutamate and glutamine resonances were fitted in order to obtain stable glutamate amplitudes. Residual, minor contributions by macromolecules are accomodated in the baseline by the fitting procedure. The applied methodology has been shown to yield maximum selectivity for the glutamate C4 resonance over glutamine and gamma-aminobutyric acid. Extensive tests yielded mean uncertainties corresponding to Cramér-Rao lower bounds with added uncertainties from background modelling [35] for the fitting of glutamate as small as 10.5% for the ACC voxel, 11.1% for the DLPFC voxel and 13.1% for the hippocampus voxel. The fitted glutamate amplitudes were corrected for different coil loading by the phantoms and the individual subject's head (principle of reciprocity) and for relaxation effects using relaxation times measured earlier [34] and assuming equal glutamate relaxation times in ACC and DLPFC. Glutamate concentrations were corrected for cerebrospinal fluid (CSF) in the voxels studied by using the csf fractions obtained by segmenting the *T*_1_-weighted images with SPM2 (www.fil.ion.ucl.ac.uk/spm/spm2.html)

### Interpersonal Reactivity Index

The Interpersonal Reactivity Index assesses each of four aspects of empathic responding, which were determined by factor analysis. We used the German translation of the IRI by Paulus [36]-‘Saarbrücker Persönlichkeitsfragebogen’ (SPF(IRI))-which was modified for altruism research. We chose three of the five 7-item-subscales measuring the core dimensions of empathy, which were answered in a 5-point Likert scale. ‘Perspective taking’ (PT) assesses the tendency to spontaneously adopt the psychological point of view of others and to reason about their mental states. The ‘empathic concern’ (EC) scale measures respondents’ prosocial feelings of warmth, compassion and concern for others. ‘Personal distress’ (PD) measures self-oriented feelings of anxiety and discomfort in response to the distress of others. The fourth subscale ‘fantasy’ (FS) taps the tendency to identify with ficticious characters in books and movies but was not used in the current investigation. Construct validity of the IRI scales was supported in several studies [2]. Internal consistencies (alpha coefficients) for the four original scales ranged from 0.71 to 0.77 [2] and for the scales of the German version from 0.58 to 0.73 [36]. Participants were asked to complete the IRI independently. Statistical analysis was performed using SPSS 14.0®.
